# Drying-mediated patterns in colloid-polymer suspensions

**DOI:** 10.1038/s41598-017-00932-z

**Published:** 2017-04-24

**Authors:** Seul-a Ryu, Jin Young Kim, So Youn Kim, Byung Mook Weon

**Affiliations:** 1 Soft Matter Physics Laboratory, School of Advanced Materials Science and Engineering, SKKU Advanced Institute of Nanotechnology (SAINT), Sungkyunkwan University, Suwon, 16419 Korea; 2 School of Energy and Chemical Engineering, Ulsan National Institute of Science and Technology (UNIST), Ulsan, 44919 Korea

## Abstract

Drying-mediated patterning of colloidal particles is a physical phenomenon that must be understood in inkjet printing technology to obtain crack-free uniform colloidal films. Here we experimentally study the drying-mediated patterns of a model colloid-polymer suspension and specifically observe how the deposit pattern appears after droplet evaporation by varying particle size and polymer concentration. We find that at a high polymer concentration, the ring-like pattern appears in suspensions with large colloids, contrary to suppression of ring formation in suspensions with small colloids thanks to colloid-polymer interactions. We attribute this unexpected reversal behavior to hydrodynamics and size dependence of colloid-polymer interactions. This finding would be very useful in developing control of drying-mediated self-assembly to produce crack-free uniform patterns from colloidal fluids.

## Introduction

Drying of a suspension droplet leaves a variety of deposit patterns of solutes^1–3^. As long as evaporation is well controlled, drying-mediated self-assembly^4^ is ubiquitously applicable to assemble nanoparticles, microparticles, polymers, proteins, and other biological molecules for applications in biological science, electronics, chemistry, and materials science^5–10^. Colloidal droplets including micro- and nanoparticles generally leave ring-like stains during evaporation, which is known as the coffee-ring effect^1, 11^. As studied, self-pinning by solute confinement at a three-phase (liquid-solid-vapor) contact region governs droplet evaporation dynamics as a basic requisite for the coffee-ring effect^12, 13^. In inkjet printing technology, the coffee-ring effect is a central problem because it obstructs production of crack-free uniform colloidal films^8, 14, 15^. Suppression of the coffee-ring effect has been an essential topic in recent decade researches^16–21^ and a variety of feasible methods for crack prevention have been suggested to date^22, 23^. A recent work shows a versatile method for crack prevention by adding a kind of short-length polymers into colloidal suspensions^24^. This method is feasible only if polymers can drive gelation of colloidal particles with short-range attraction^25^, so called gelation-driven crack prevention^23^. Nonadsorbing polymers are useful for driving gelation^23^. Importantly, driving weak gelation by polymer addition into colloidal suspensions would be dependent upon particle size and polymer concentration^26, 27^, which has long been studied in terms of phase separation and gelation in model colloid-polymer mixtures^28–30^. For better control of drying-mediated self-assembly in colloidal suspensions, a deep understanding for hydrodynamics and size dependence of colloid-polymer interactions is increasingly warranted for large-area, highly ordered, and crack-free uniform colloidal films.

**Figure 1:**
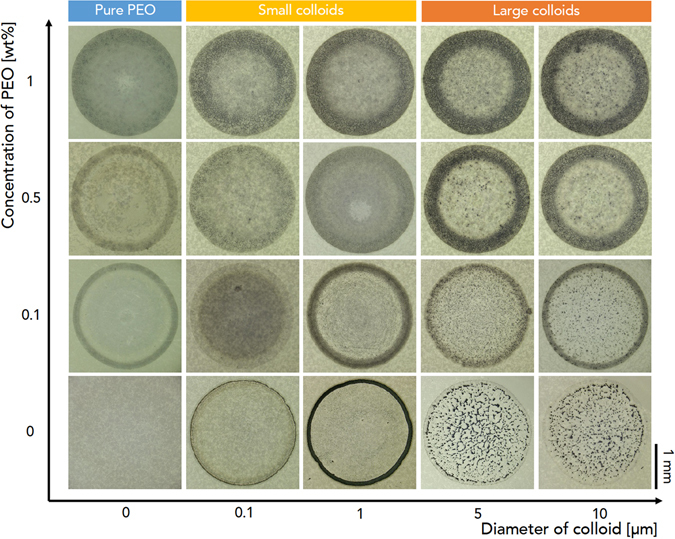
A phase diagram for drying-mediated patterns. Deposit patterns were taken by varying colloid size (100 nm ~ 10 *μ*m in diameter) and polymer concentration (0~1.0 wt%) in a model colloid-polymer system, consisting of poly(methyl methacrylate) (PMMA) (PMMA concentration is fixed as 0.1% (1 mg/ml)) colloid and poly(ethylene oxide) (PEO) polymer (the viscosity average molecular weight *M*_*v*_ = 1,000 kg/mol) suspended in water. Deep interpretation about patterns is described in the text.

In this work, we demonstrate an experimental study on how deposit patterns emerge from evaporating droplets of colloid-polymer suspensions by varying particle size and polymer concentration. We obtain a phase diagram for particle size and polymer concentration utilizing a model colloid-polymer system. The model suspension without polymers shows typical deposit patterns: small colloids tend to make rings and large colloids make bumps, as can be seen in Fig. 1. Our important finding for colloid-polymer suspensions is that at high polymer concentrations, ring-like patterns appear in suspensions with large colloids, contrary to suppression of ring formation in suspensions with small colloids thanks to colloid-polymer interactions. This discrepancy is unexpected and important in controlling the final deposit patterns. We discuss the underlying mechanism for the reversal deposit patterns in terms of hydrodynamics and size dependence of colloid-polymer interactions.

## Results

### A phase diagram for drying-mediated patterns

We experimentally observed deposit patterns with varying colloid size (100 nm ~ 10 *μ*m in diameter) and polymer concentration (0~1.0 wt%) from a model colloid-polymer system (see the Experiments), consisting of poly(methyl methacrylate) (PMMA, purchased from Phosphorex) colloid and poly(ethylene oxide) (PEO) polymer with the viscosity average molecular weight *M*_*v*_ = 1,000 kg/mol (Sigma Aldrich 372781) suspended in water. Here the PMMA concentration was fixed as 0.1% (1 mg/ml). Direct visualization of deposit pattern formation, as demonstrated in Fig. 1, was taken with a digital microscope (VH-Z100R, Keyence) at a rate of 15 frame per second. The left column of Fig. 1 demonstrates the deposit patterns for pure polymer solutions without colloids (as marked by pure PEO). As the polymer concentration increases, the deposit pattern has dim rings. This result is consistent with typical deposit patterns taken from polymer suspended droplets^31^: no stains result from de-pinning dynamics and rings from pinning dynamics^23, 32^. The middle two columns of Fig. 1 demonstrate the suppressed coffee-ring effect for colloid-polymer suspensions with small colloids (as marked as small colloids). Small colloids originally make rings at the perimeter in the absence of polymers, while they gradually become left randomly inside the droplet as nonadsorbing polymers are increasingly added into colloidal suspensions. This behavior is well studied as the suppression model^23^. The right two columns of Fig. 1 demonstrate the unexpected result from colloid-polymer suspensions with large colloids (as marked as large colloids). The right bottom two images show a typical result from evaporative patterns for large colloids: bump-like stains are formed randomly inside the droplet^11^. However, as the polymer concentration increases, large colloids move toward the perimeter, resulting to formation of ring-like patterns, which is unexpected yet very convincing.

### Reverse behaviors for small and large colloids

We were able to observe the reversal deposit patterns particularly taken from small (100-nm-diameter) and large (10-*μ*m-diameter) colloids, as demonstrated in Fig. 1. As well known, small colloids tend to move toward the perimeter in the absence of polymers, leaving ring-like patterns^16^, while they stay at the whole part of the droplet, as polymer is added, which is known as the suppressed coffee-ring effect^18^. Contrary to small colloids, large colloids show the reverse deposit behaviors: they tend to stay randomly inside the droplet in the absence of polymers, making bump-like patterns, while they move toward the perimeter as polymer is added.

**Figure 2:**
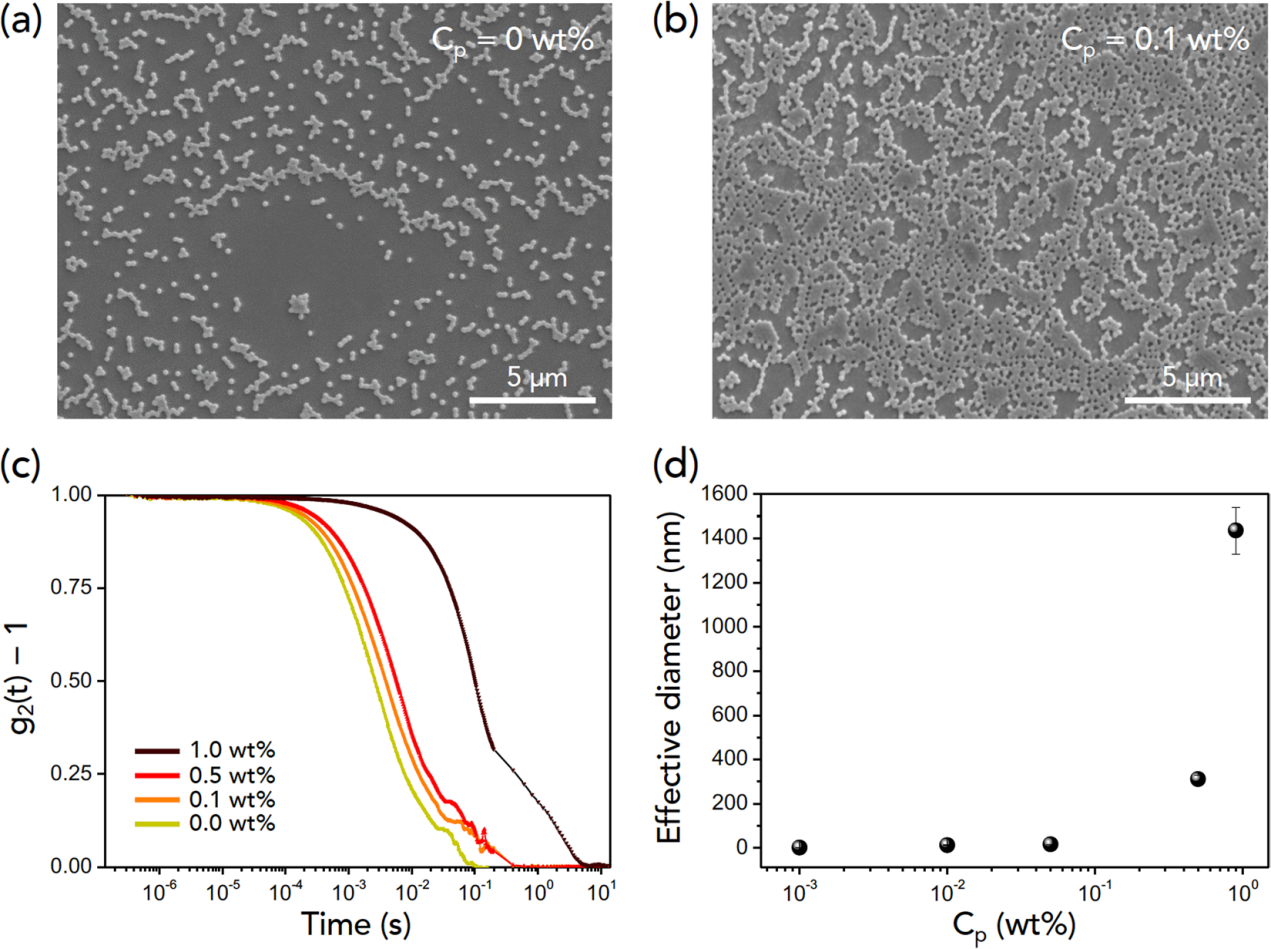
Colloid-polymer interactions for small colloids. (**a**,**b**) SEM images for the center of the dried droplet with small (100-nm-diameter) colloids without polymers (*C*_*p*_ = 0 wt%) and with polymers (*C*_*p*_ = 0.1 wt%). (**c**,**d**) Light scattering experiments. The intensity autocorrelation function *g*_2_(*t*) − 1 versus time, taken from the diffusing wave spectroscopy (DWS), shows attractive interactions between colloids and polymers. The effective diameter, taken from the intensity average diameter of the dynamic light scattering (DLS), increases with the polymer concentration *C*_*p*_.

The suppressed coffee-ring effect for colloid-polymer suspensions made of small colloids is a consequence of interplay between hydrodynamics and colloid-polymer interactions (particularly the colloid flocculation can be induced by the depletion effect of polymer chains^31^). In the absence of polymers, coffee-ring-induced hydrodynamics determines evaporation and deposit dynamics, resulting in the ring-like deposit patterns. As the polymer concentration increases, polymer-induced attraction becomes predominant^33, 34^. Polymers between small colloids are able to weakly attract neighboring colloids and then enable neighboring colloids to assemble as clusters, as seen in Fig. 2(a,b), taken from scanning electron microscopy (SEM). It is known that adsorbing polymer chains can induce the aggregation of colloidal particles during evaporation^31^. The resulting colloidal clusters of small colloids tend to stay at the middle of the droplet despite the existence of the edge-ward coffee-ring flow^35^. Evidently the suppressed coffee-ring behavior of small colloids by polymer addition is seen from direct visualization of droplet evaporation of 100-nm-diameter PMMA colloids without PEO polymer (Supplementary Movie 1) and with PEO polymer (Supplementary Movie 2), as consistent with other observations^18^.

The colloid-polymer interactions for small colloids were investigated with light scattering experiments. The intensity autocorrelation function *g*_2_(*t*) − 1 versus time in Fig. 2(c), taken from the diffusing wave spectroscopy (DWS), demonstrates that upon addition of the PEO polymer, the autocorrelation decays more slowly, which is probably as a result of polymer-induced aggregation of colloids. The present analysis is consistent to the previous work^23^. The slowed relaxation of the particle dynamics can be attributed to the aggregation of colloids or the formation of particle clusters. The effective diameter in Fig. 2(d), obtained from the dynamic light scattering  (DLS), also increased with adding PEO polymers. The increased effective particle size resulted from the increased average size of particle clusters.

**Figure 3:**
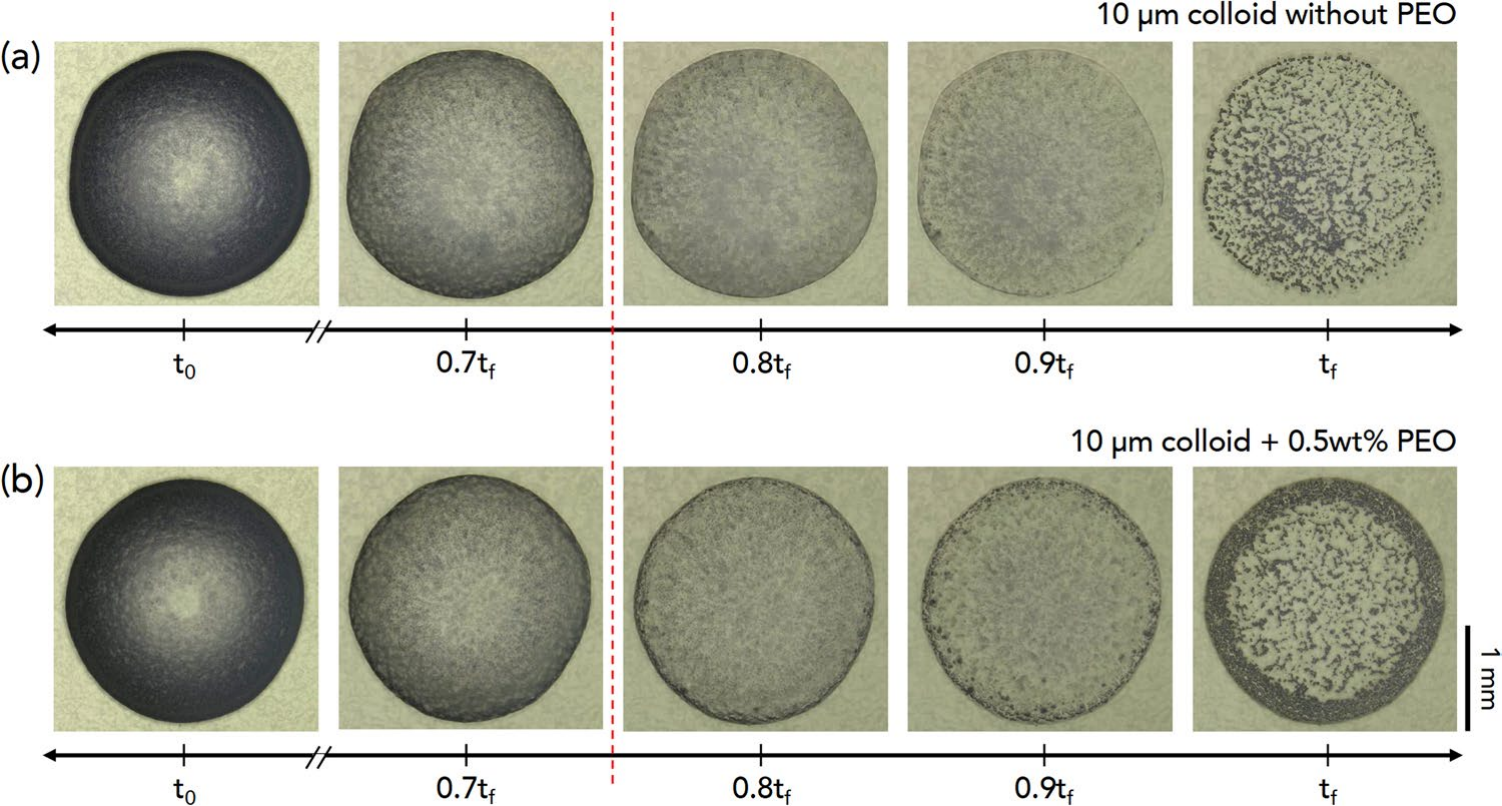
Evaporation evolution for large colloids. Real-time observations for evaporation evolution of large (10-*μ*m-diameter) colloids (**a**) without polymers and (**b**) with polymers. The evaporation time *t* is normalized by the complete drying time *t*_*f*_. Evaporation behaviors look similar before 0.8*t*_*f*_ but become significantly different after 0.8*t*_*f*_: large colloids tend to stay randomly inside the droplet, leaving the bump-like pattern at *t*_*f*_ of (**a**), while they move toward the perimeter, leaving the ring-like pattern at *t*_*f*_ of (**b**).

The reverse pattern formation for colloid-polymer suspensions with large colloids as found in Fig. 1 is presumably relevant to the size dependence of the aggression dynamics of colloids with polymer additives^18, 31^. With adding polymers with identical molecule weights, small colloids are more favorable to undergo aggregation than large ones^24, 26^. This consideration implies that colloid-polymer interactions for large colloids would become very weakened and coffee-ring-induced hydrodynamics would become predominant for large colloids, regardless of polymer addition. We compared the evaporation evolution without and with polymers for large colloids, as seen in Fig. 3, where the evaporation time *t* is normalized by the complete drying time *t*_*f*_. Interestingly, evaporation behavior looks similar before 0.8*t*_*f*_ but becomes significantly different after 0.8*t*_*f*_. Large colloids tend to stay randomly inside the droplet, leaving the bump-like pattern at *t*_*f*_ without polymer, while they move toward the perimeter, leaving the ring-like pattern at *t*_*f*_ with polymer (Supplementary Movies 3 and 4). On this basis, we are able to suppose that hydrodynamics for the final period of evaporation (0.8*t*_*f*_) would be essential to understand the reversal pattern formation for large colloids.

## Discussion

Pattern formation from evaporating colloidal suspensions is an important issue in soft matter science and inkjet printing technology. Since the coffee-ring effect is a central problem that obstructs crack-free uniform colloidal films, the suppressed coffee-ring effect has been extensively studied. Recently it was reported that polymer addition into colloidal suspensions could effectively suppress the coffee-ring effect and induce gelation for crack prevention^23^. A full understanding of polymer addition into colloidal suspensions is still required for better control of the coffee-ring effect. In the present work, we show how pattern formation of colloidal suspensions can be modified with polymer addition. A phase diagram in terms of particle size and polymer concentration is demonstrated in Fig. 1. The underlying mechanism about the suppressed coffee-ring behavior for small colloids can be explained with the polymer-induced aggregation of small colloids in Fig. 2(c,d). To understand the final deposit patterns of colloid-polymer suspensions for large colloids, we need to consider the roles of polymer additives and coffee-ring-driven hydrodynamics.

What is the role of polymer addition in colloid-polymer suspensions with large colloids? As mentioned, polymers would not significantly contribute to attraction between large colloids, but they can make the contact line strong. This possibility appears in pure polymer suspensions without colloids in Fig. 1. The addition of sufficient amounts of polymers would be favorable to let the droplet to get pinned strongly. As the ring grows, the ring width would increase^2^. This consequence would make the evaporation rate of the droplet for large colloids with polymers to slow down and the lifetime (equivalently *t*_*f*_) becomes longer than that for the droplet in case of no polymers.

**Figure 4:**
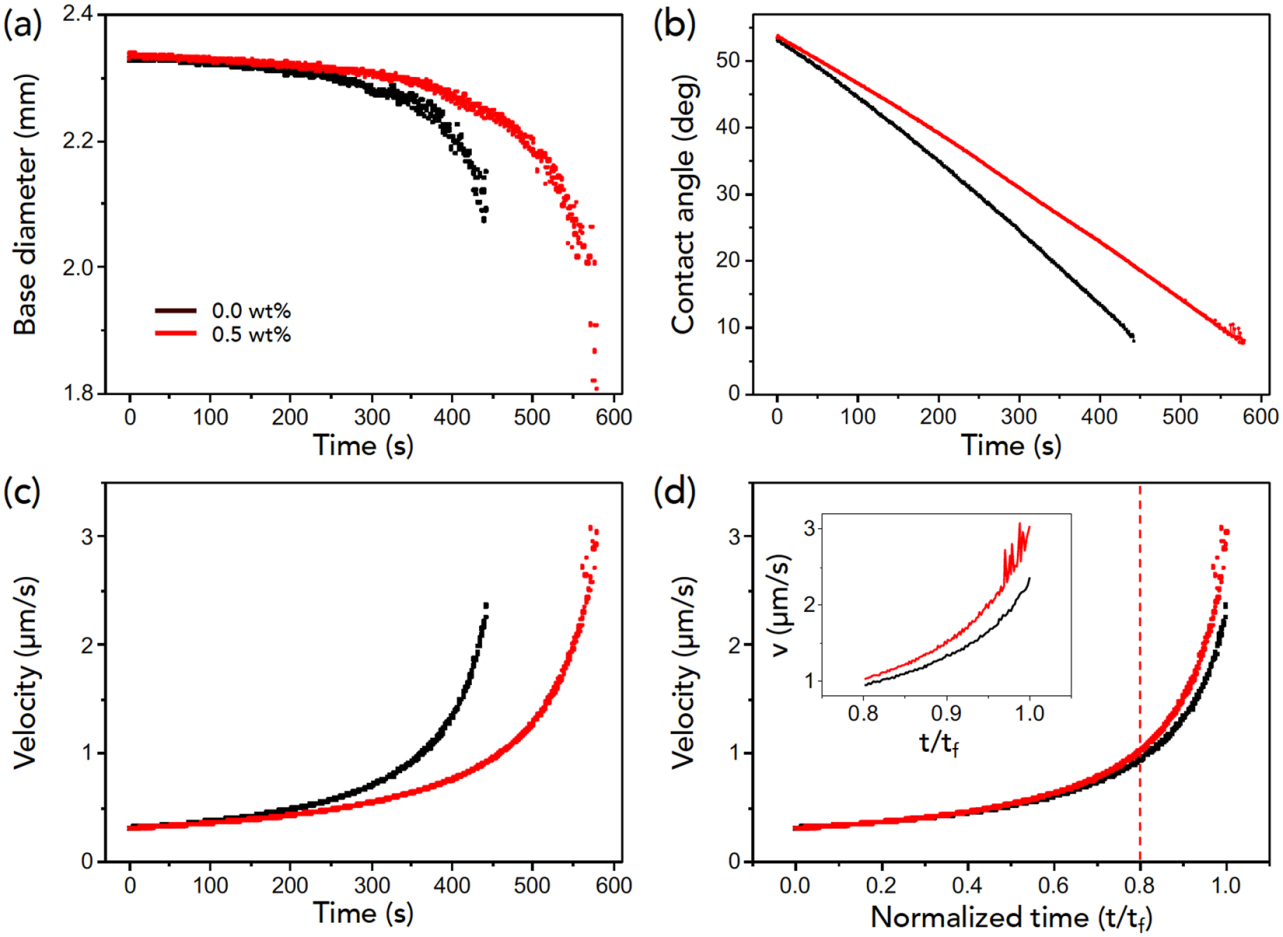
Hydrodynamics of droplet evaporation with large colloids. (**a**,**b**) Time evolution of the base diameter and of the contact angle taken for suspension droplets of large (10-*μ*m-diameter) colloids without and with polymers. The complete drying time of the droplet with large colloids is slower with polymers than without polymers: therefore, the base diameter and the contact angle more slowly decrease with time with polymers than without polymers. (**c**,**d**) Time evolution of estimated height-averaged radial velocity, taken from ref. 36 and data in (**a**,**b**). Here (**d**) is rescaled from (**c**) with the normalized time *t*/*t*_*f*_ for comparison. Inset indicates the velocity details after 0.8*t*_*f*_.

The height-averaged radial velocity of flows is given as $\bar{u}=\frac{{D}^{\ast }}{\theta (t)}\cdot \frac{1}{\sqrt{R(R-r)}}$ with ${D}^{\ast }=2\sqrt{2}{D}_{va}{\rm{\Delta }}c/(\pi \rho )$ (where *D*_*va*_ is the diffusion constant of vapor in air, Δ*c* is the vapor concentration difference between the drop surface and the surroundings, *ρ* is the liquid density, *θ*(*t*) is the time-dependent contact angle, *R* is the drop radius, *r* is the distance from the contact line (fixed to be *r* = 0.5 mm for analysis), and (*R* − *r*) indicates the distance from the drop center^36^). Considering materials constants for water^36^ and our observations for 2*R* [Fig. 4(a)] and *θ* [Fig. 4(b)] from side-view images of the evaporating droplet (Supplementary Movies 5 and 6), we estimated the $\bar{u}$ value as seen in Fig. 4(c,d). Here the most important finding is that at the final period of evaporation in the inset of Fig. 4(d), the velocity of suspensions with large colloids and polymers is slightly faster than that in the absence of polymers. Probably, the faster velocity would enhance the rush behavior of large colloids toward to the perimeter, as observed in Fig. 3.

**Figure 5:**
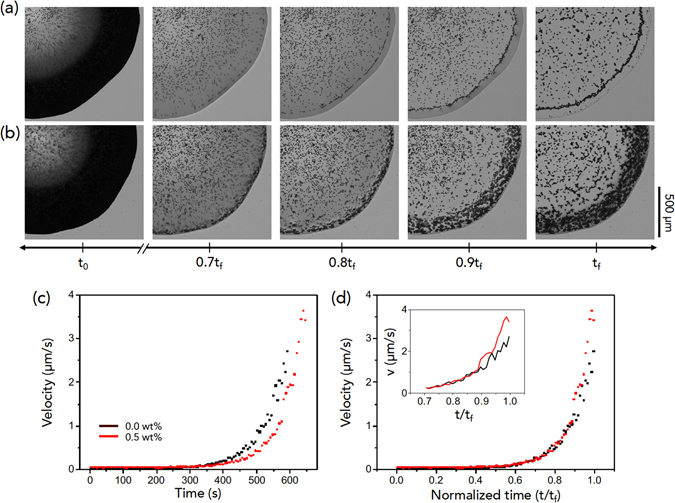
Flocculation and hydrodynamics with large colloids. (**a**,**b**) Verifying the difference in evaporation evolution for 10-*μ*m-diameter colloids without and with polymers. Time evolution of the bottom-view image of droplet evaporation was taken with an inverted microscope. (**c**,**d**) Radial velocity of large colloids, taken at the position of 0.5 mm from the contact line of the droplet. Here (**d**) is rescaled from (**c**) with the normalized time *t*/*t*_*f*_ for comparison. Inset indicates the velocity details after 0.7*t*_*f*_.

The bottom-view visualization of droplet evaporation of 10-*μ*m-diameter PMMA colloids without PEO polymer (0.0 wt%) and with PEO polymer (0.5 wt%) was taken with an inverted microscope, as demonstrated in Fig. 5(a,b) (Supplementary Movies 7 and 8). The bottom radial velocity of flows was measured and summarized in Fig. 5(c,d), which are consistent with Fig. 4(c,d). The good agreement between estimations [Fig. 4(c,d)] and experiments [Fig. 5(c,d)] suggests that the long lifetime and the rapid rush behavior for suspensions with large colloids and polymers would enhance the ring-like pattern formation at the final period of evaporation.

Finally, we discuss three characteristic time scales in the process: adsorption of polymers on the colloid surface, kinetics of bridging flocculation, and evaporation dynamics. The polymer adsorption would be stabilized by holding the mixed suspensions for a day before experiments. Presumably bridging flocculation and evaporation dynamics would simultaneously depend on the flow patterns during evaporation. The number concentration must be different at a fixed concentration for small and large colloids. Practically at a few wt% of colloids, instantaneous adsorption of polymers is expectable^37^. Most importantly, smaller colloids are more favorable for flocculation^38^, presumably induced by their higher number concentration. Flocculation for small colloids would contribute to the suppressed coffee-ring formation through gelation^23^, while that for large colloids ironically facilitates the typical coffee-ring formation. As demonstrated in Fig. 5(a), there is no flocculation for large colloids without polymers, showing the reverse coffee-ring effect at the final period of evaporation (0.8*t*_*f*_)^16^. However, flocculation takes place among large colloids with addition of polymers at the final period of evaporation (0.8*t*_*f*_), as shown in Fig. 5(b), which delivers large colloids to the contact line and eventually leaves the coffee-ring patterns. Consequently, the drying-mediated deposit patterns would be a consequence of cooperation in flocculation and evaporation.

In conclusion, our experimental study demonstrates that polymer addition in colloid-polymer suspensions as model ink materials would significantly contribute to formation of final deposit patterns with intervention of coffee-ring-driven hydrodynamics and size-dependent colloid-polymer interactions. We show unexpected reversal pattern behaviors between small and large colloids with or without polymer additives. Our finding would be useful for a deep understanding for the importance of hydrodynamics and colloid-polymer interactions that would be essential for production of large-area, highly ordered, and crack-free uniform colloidal films.

## Experiments

### Materials

As model colloids, poly(methyl methacrylate) (PMMA) colloidal particles were used. PMMA microspheres were originally purchased from Phosphorex with density of 1.19 g/cm^3^ and diameter of 100 nm, 1 *μ*m, 5 *μ*m, and 10 *μ*m on average (the particle size of 100 nm and 1 *μ*m was checked by SEM images and that of 5 *μ*m and 10 *μ*m by a digital microscope). The microspheres were originally supplied as 1% solid suspensions (10 mg/ml) and suspended in de-ionized (DI) water containing a small amount of surfactant and 2 mM of sodium azide as an anti-microbial agent. For all experiments, the initial concentration of colloids was fixed to be dilute as 0.1% (1 mg/ml) by adding pure water obtained from DI water system (ELGA). Poly(ethylene oxide) (PEO) was used as model polymers and purchased from Sigma-Aldrich (372781-250G) with the viscosity average molecular weight *M*_*v*_ = 1,000 kg/mol. By mixing PEO into water and colloidal suspensions with vortex mixer for 30 minutes, we prepared various colloid-polymer suspensions for experiments. These suspensions were hold for 24 hours and then used in experiments with stabilized suspensions. Microscope cover glasses with 24 × 50 mm (Deckglaser) were used as flat solid substrates. All substrates were cleaned for 10 minutes with ultrasonic cleaner (UC-10, Lab Companion) and dried under blow gun.

### Evaporation experiments

For visualization of droplet evaporation, the initial drop volume was controlled to be 1 *μ*l. Prior to evaporation, a 1-*μ*l-volume droplet was delivered from a micro pipette on the substrate. The top-view image of droplet evaporation for Figs 1 and 3 was taken with a digital microscope (VH-Z100R, Keyence, Japan) at a rate of 15 frame per second. The side-view image of droplet evaporation, particularly for measurement of base diameter and contact angle in Fig. 4, was taken with a drop shape analyzer (DSA25, Krüss, Germany) at a rate of 25 frame per second. The bottom-view image of droplet evaporation for Fig. 5 was taken with an inverted microscope (CKX53, Olympus, Japan) at a rate of 0.5 frame per second. By analyzing each image using ImageJ software, the average velocity of colloids was measured at the position of 500 *μ*m from the contact line of an evaporating droplet. All evaporation experiments were conducted at temperature of 24–26 °C and relative humidity of 45–52%. Evaporation experiments for each condition were repeated five times.

### Light scattering experiments

DWS experiments in Fig. 2(c) were performed on a DWS RheoLab II (LS Instruments) in transmission mode to obtain the autocorrelation function of the dense colloid systems. The coherent source was a diode laser (with wavelength *λ* = 658 nm and the power 30 mW). The samples were measured at 25 ± 0.02 °C. DLS experiments in Fig. 2(d) were performed with the BI-200 SM goniometer (Brookhaven Instruments) using an EM-9865 photomultiplier and a digital correlator (BI-9000AT) for measurement of the intensity average diameters of colloids with varying polymer concentrations. DWS experiments were repeated eight times for each condition.

## Electronic supplementary material

Movie 1

Movie 2

Movie 3

Movie 4

Movie 5

Movie 6

Movie 7

Movie 8

Supplementary file
