# Supplementary material for: Drying-mediated patterns in colloid-polymer suspensions
Source: Sci Rep. 2017 Apr 24;7:1079. doi: 10.1038/s41598-017-00932-z (PMC5430651; doi:10.1038/s41598-017-00932-z)
Supplement: Supplementary file 9 — Supplementary file [file 41598_2017_932_MOESM9_ESM.doc]

Supplementary Information

Drying-mediated patterns in colloid-polymer suspensions

Seul-a Ryu,^1^ Jin Young Kim,^1^ So Youn Kim,^2^ & Byung Mook Weon^1,*^

^1^ Soft Matter Physics Laboratory, School of Advanced Materials Science and Engineering, SKKU Advanced Institute of Nanotechnology (SAINT), Sungkyunkwan University, Suwon 16419, Korea

^2^ School of Energy and Chemical Engineering, Ulsan National Institute of Science and Technology (UNIST), Ulsan 44919, Korea

*Corresponding author: bmweon@skku.edu

Supplementary Video Legends:

**Movie 1:** Top-view visualization of droplet evaporation of 100-nm-diameter PMMA colloids without PEO polymer (0.0wt%), taken with a digital microscope, for Fig. 1.

**Movie 2:** Top-view visualization of droplet evaporation of 100-nm-diameter PMMA colloids with PEO polymer (0.5wt%), taken with a digital microscope, for Fig. 1.

**Movie 3:** Top-view visualization of droplet evaporation of 10-µm-diameter PMMA colloids without PEO polymer (0.0wt%), taken with a digital microscope, for Fig. 3(a).

**Movie 4:** Top-view visualization of droplet evaporation of 10-µm-diameter PMMA colloids with PEO polymer (0.5wt%), taken with a digital microscope, for Fig. 3(b).

**Movie 5:** Side-view visualization of droplet evaporation of 10-µm-diameter PMMA colloids without PEO polymer (0.0wt%), taken with a drop shape analyzer, for Figs. 4(a) and 4(b).

**Movie 6:** Side-view visualization of droplet evaporation of 10-µm-diameter PMMA colloids with PEO polymer (0.5wt%), taken with a drop shape analyzer, for Figs. 4(a) and 4(b).

**Movie 7:** Bottom-view visualization of droplet evaporation of 10-µm-diameter PMMA colloids without PEO polymer (0.0wt%), taken with an inverted microscope, for Figs. 5(a), 5(c), and 5(d).

**Movie 8:** Bottom-view visualization of droplet evaporation of 10-µm-diameter PMMA colloids with PEO polymer (0.5wt%), taken with an inverted microscope, for Figs. 5(b), 5(c), and 5(d).
